# Country and gender differences in the association between violence and cigarette smoking among youth

**DOI:** 10.1186/s13031-020-00332-7

**Published:** 2020-12-14

**Authors:** Niveen M. E. Abu-Rmeileh, Ethel Alderete, Abdullatif Husseini, Jennifer Livaudais-Toman, Eliseo J. Pérez-Stable

**Affiliations:** 1 Institute of Community and Public Health- Birzeit University, West Bank, Occupied Palestinian Territory (oPt), Birzeit, Palestine; 2 Consejo Nacional de Investigaciones Científicas y Técnicas (CONICET), Universidad Nacional de Jujuy, Instituto de Ciencia y Tecnología Regional (ICTER), Jujuy, Argentina; 3 Division of General Internal Medicine, Department of Medicine, at the University of California, San Francisco, USA; 4 Division of Intramural Research, National Heart, Lung and Blood Institute, National Institutes of Health, Bethesda, USA

**Keywords:** VIOLENCE, SMOKING, YOUTH, GENDER, CROSS-COUNTRY

## Abstract

**Background:**

Exposure to violence in youth may be associated with substance use and other adverse health effects. This study examined cigarette smoking in two middle-income areas with different levels and types of exposure to violence.

**Methods:**

Association of exposure to verbal and physical violence with cigarette smoking in the West Bank oPt (2008) and in Jujuy Argentina (2006) was examined using cross-sectional surveys of 14 to 17-year old youth in 7th to 10th grade using probabilistic sampling.

**Results:**

Violence exposure rates were more than double for Palestinian girls (99.6% vs. 41.2%) and boys (98.7% vs. 41.1%) compared with Argentinians. The rate of current cigarette smoking was significantly higher among Argentinian girls compared with Palestinian girls (33.1% vs. 7.1%, *p* < 0.001). Exposure to verbal violence from family and to physical violence increased the odds of current cigarette smoking, respectively, among Argentinian girls (aOR = 1.3, 95% CI = 1.0–1.7; aOR = 2.5, 95%CI = 1.7–3.8), Palestinian girls (aOR 2.2, 95%CI = 1.1–2.4; aOR = 2.0, 95%CI = 1.1–3.6) and Argentinian boys (aOR = 1.5, 95%CI = 1.1–2.0; aOR = 2.2, 95%CI = 1.6–3.0), but not among Palestinian boys.

**Conclusion:**

Findings highlight the importance of producing context and gender specific evidence from exposure to violence, to inform and increase the impact of targeted smoking prevention strategies.

## Introduction

Expert reports express concern over increasing or persistently high smoking prevalence in low- to middle Human Development Index (HDI) countries. Another major concern is an increase in smoking prevalence among youth, particularly among girls [1, 2]. Although the lack of full implementation of tobacco control policies in many countries undermines global tobacco control efforts [1], the complex interplay of bio-social-environmental factors that promote smoking in subgroups of youth with different socioeconomic, cultural and contextual characteristics are not sufficiently understood. This gap in knowledge sets limits to the development of prevention tools with effectiveness in addressing diversity within and across countries.

Risk factors associated with youth smoking like peer and media influences, have been consistently identified across countries from varied HDI levels [3–9]. Evidence supports the role of social norms that may influence substance use behavior, including smoking, with traditional gender roles usually acting as protectors especially among females [10–12]. However, the influence of stress inducing personal, family and contextual factors like exposure to violence is not sufficiently understood. The role of stress on substance use has been analyzed on the basis of several conceptual frameworks like the stress and coping theory [13], the social learning theory [14] and the tension reduction theory [15]. Youth in most countries are likely to be exposed to violent events either by interpersonal aggression, local gangs, organized crime, institutionalized violence and armed conflicts or wars, in addition to exposure to violence within the family and school settings [16, 17]. Violence refers to the intentional use of force or power, threatened or actual, against oneself, another person, or against a group or community that either result in or has a high likelihood of resulting in injury, death, or psychological harm [18]. Experiencing violent events has been shown to be associated with behavioral risk factors like substance use disorders and psychiatric morbidity [19, 20].

Several studies have been conducted among school-based populations to assess the relationship between a reduced set of violent events and cigarette smoking. In these studies, from China, Malawi, the United States, Chile, and the Western Pacific, bullying or being involved in physical fights was significantly associated with cigarette use [21–25]. A cross-country school-based study showed a consistent association between physical fights and cigarette smoking in 6 countries within the Western Pacific [26]. Another cross-country study used a community-based sample of youth in five urban sites (Baltimore, New Delhi, Johannesburg, Ibadan, and Shanghai) to assess the association of neighborhood contextual factors with smoking behavior, and victimization measured as being pushed or shoved, hurt in a fight, verbally threatened, threatened with a weapon, or hurt with a weapon. Not all neighborhood-level factors were significant across sites. Witnessing community violence showed more consistency, being positively associated with ever smoking and victimization in New Delhi and Johannesburg [27]. Regarding gender differences, two school based population studies in the US reported inconsistent results [23, 25].

Country level social, economic and/or political processes may modulate how youth experience and process exposure to violence at the personal level, conditioning behavioral responses to stress [28–30]. This study examined the association of exposure to verbal and physical violence from family and non-family members with cigarette smoking, among middle school-age youth from the West Bank oPt and the Province of Jujuy, Argentina. The study adds to the scarce literature on cross-country studies of violence and smoking among school-age youth and adds a gender perspective to assess differences between girls and boys.

### Study sites

Violence associated with economic crises, inequality, social conflicts and military dictatorships has been a chronic feature of many Latin Americans countries. Contributing local phenomena include state repression, impoverishment with social marginalization, and delinquency and crime linked to the illicit drug trade [31]. The Province of Jujuy in Argentina (population of 673,307) is socioeconomically disadvantaged with human development indicators significantly lower than national averages [32, 33]. In Argentina the national average household poverty rate reached a peak of 54% in 2002 after the 2001 economic crisis  [34, 35].

The West Bank oPt presents unique features defined by regulatory and trade policies and Israeli control, including mobility restrictions within the territory [36, 37]. Palestinians in the West Bank have coexisted with armed conflicts for more than 70 years witnessing wars, displacements and uprisings [38, 39]. Of the West Bank, oPt governorates, Ramallah located in the central region with a population of 279,730 is the provisional site of the Palestine National Authority, as well as a hub for business and donor agencies. Jenin is an important governorate of the north, with a population of 256,619 and is located in the main agricultural region. Ramallah’s localities are predominantly much more prosperous compared to Jenin [36]. In 1997, the household poverty rate in the West Bank averaged 25.3% and had increased to 29.2% by 2017 [37, 40]. Geographic variations showed that poverty rate was 53% around Jenin, and less than 10% in Ramallah [37].

Rates of ever smoking cigarettes among youth were similar in the West Bank and Argentina, 50% and 55% between 2000 and 2001, but current cigarette smoking was more prevalent in Argentina than in the West Bank (25% vs. 14%) [41]. The two research sites in the West Bank, oPt, Ramallah and Jenin, had similar current cigarette smoking rates among youth, 17% and 20% respectively in 2008 [42]. While current cigarette smoking rates by sex do not differ significantly in Argentina (20.2% vs. 21.7%) [43], rates are significantly lower among Palestinian girls than boys (4% vs. 32%) [42].

A study of exposure to violent events from military and settlers among 10th and 11th grade students in Ramallah in 2003, showed that 80% had seen shootings, 28% had seen a stranger killed, and 11% had seen a friend or neighbor killed [44]. In 2008 in Ramallah and Jenin, among 7th to 10th grade students, 58% were victims of violence related to actions from the armed forces, 67% witnessed violence, 56% were beaten by school personnel and 44% were beaten by parents [42]. Among Palestinian parents, support for physical punishment decreases with education and income level [45]. In Argentina, violent deaths among adolescents increase with increased poverty [46]. According to UNICEF, almost 70% of parents in Argentina reported the use of verbal or physical violence to discipline their children [47]. In a study among middle school students, 56% had witnessed or were subjects of more than 10 acts of violence, 58% witnessed someone being arrested with violence, and 50% knew someone who had been beaten by a non-family member [48] .

## Methods

### Palestinian sample

A cross-sectional self-administered survey was conducted in two governorates of the West Bank (Ramallah and Jenin) in the year 2008 targeting students in the 7th, 8th, 9th and 10th grades. The sample was selected using single stage cluster sampling, with the class section as the primary sampling unit. The number of students was allocated equally between the two governorates, 1500 students in each governorate. Given an average number of 52 students in each section, the number of students participating in the survey was obtained by sampling 47 schools in Ramallah and 45 schools in Jenin. Due to nonresponse, 3 schools were added to the sample in Ramallah and 5 in Jenin, yielding 80% public schools, 10% United Nations refugee relief schools and 4% private schools. All students in the selected sections were included and a total of 3107 participated. For this analysis, 1627 students 14 to 17 years of age were included. The research protocol was approved by the review committee of the Institute of Community and Public Health, Birzeit University. The consent was granted by the school principals who informed the parents that they approved the study.

### Argentinian sample

A longitudinal study was conducted in the Province of Jujuy in Northwest Argentina. The sample was selected using a probabilistic multi-stage cluster sampling design with middle schools as the primary sampling units [43]. The baseline data were collected in 2004 among all enrolled 8th graders in the 24 public and 3 private selected schools through a self-administered questionnaire. Three follow up surveys were conducted between 2005 and 2007. Data related to violence exposure was collected in 2006 and for this analysis we included respondents 14 to 17 years of age (*N* = 2897). The UCSF Committee on Human Research and an NIH-certified human subjects research board in Buenos Aires based at Centro de Educación Médica e Investigaciones Clínicas (CEMIC) approved the research protocol. Passive consent was requested from caretakers and students signed an active consent.

### Measures

The questionnaires used in the two countries had similar comparable sociodemographic, smoking behavior and violence exposure questions which were used in this study, in addition to country specific questions. The two studies were conducted in the framework of international tobacco control research funding initiatives.

Questions used in this analysis were sex and age of the students, and the highest education level and the occupational status of the parents or primary caregiver. Students reported on their perception of their own health status categorized as excellent, very good, good, average, or below average. Students were asked if they felt nervous or anxious, and if they felt down or sad, with response categories of yes or no.

Questions about the smoking status of their father or mother, their siblings, and teachers with answers categorized as binary variables, yes or no, were included. The students also responded to the questions “Do you think that you will smoke a cigarette in the next year?”, “Do you think that you would be able to stop smoking if you wanted?” categorized as yes/no, and to the question “If a friend offers a cigarette, would you smoke?” categorized as definitely no, probably no, probably yes, definitely yes. Smoking behavior was assessed by age when first tried cigarettes, smoking cigarettes in the past month, number of days when cigarettes were smoked in the last month and number of cigarettes smoked in the days when cigarettes were smoked in the last month.

Exposure to violence was assessed based on several questions regarding verbal and physical violence. Verbal violence from family members referred to being insulted by family members in the Argentina sample and to being insulted by parents or siblings in the Palestine sample. Physical violence from family members referred to being hit by family members in Argentina and to being beaten by parents or siblings in the Palestine sample. Physical violence from nonfamily members referred to being injured by a knife, blade or gun, or to being hit during a robbery in the Argentina sample, and to being exposed to shooting by live or rubber bullets, being exposed to gas bombs or being beaten by Israeli soldiers, in the Palestine sample. Any physical violence included physical violence from family and nonfamily members as defined in the disaggregated variable. Any verbal or physical violence from any source included the sum of all violence exposure variables.

### Statistical analysis

The statistical program Stata (version 14.2) was used for data analysis [49]. The sampling design for each population was incorporated into all analyses by specifying geographic areas as strata and schools as clusters (primary sampling unit), as well as including weights to adjust for disproportionate stratification. Because data from two discrete populations were combined, sampling weights within each population were rescaled so that the sum of the weights, within each population, was equal to the sample size. Standard errors and confidence intervals were estimated via the Taylor expansion approximation (*svy* prefix command in Stata version 14.2). We conducted descriptive analyses examining the distribution of demographic characteristics by country (Table 1) and the distributions of smoking behaviors (Table 2) and exposure to violence (Table 3) by country, within each gender. Chi-square tests and *p* values were calculated.

**Table 1:** Demographics of adolescents 14–17 years old by study site: Jujuy, Argentina in 2006 and the oPt in 2008 (total *N* = 4524)

	Argentina ***N*** = 2897 N (%)*	oPt ***N*** = 1627 N (%)*	***p***-value
**Gender**			0.040
Girls	1575 (54.9)	735 (45.0)	
Boys	1322 (45.1)	892 (55.0)	
**Age (mean ± SE)**	15.4 ± 0.05	14.7 ± 0.05	< 0.001
**Age**			< 0.001
14–15 years	1948 (68.8)	1360 (83.5)	
16–17 years	949 (31.2)	267 (16.5)	
**Highest Education of Father/Primary Provider**			0.140
Less than high school	1523 (55.4)	699 (47.6)	
High school or more	1364 (44.6)	770 (52.4)	
**Occupational Status of Father/Primary Provider**			0.003
Not working	343 (11.5)	240 (16.1)	
Working Full or Part-time	2542 (88.5)	1260 (83.9)	
**Father or Mother are smokers**	1359 (47.8)	903 (56.8)	< 0.001
**Brothers or sisters are smokers**	934 (33.2)	507 (32.3)	0.659
**Teachers smoke at school**	1846 (67.6)	953 (64.4)	0.519
**If a friend offers a cigarette, would you smoke?**			< 0.001
Definitely no/Probably no	1809 (61.4)	1331 (81.8)	
Definitely yes/Probably yes	1069 (38.6)	295 (18.2)	
**Do you think that you will smoke a cigarette in the next year?**			< 0.001
Definitely no/Probably no	1621 (55.5)	1271 (78.2)	
Definitely yes/Probably yes	1257 (44.5)	356 (21.9)	
**How do you evaluate your health status?**			< 0.001
Less than very good/excellent	1764 (59.0)	277 (17.1)	
Very good/excellent	1127 (41.0)	1350 (82.9)	
**Felt tense, nervous or anxious**^**a**^	871 (30.4)	1157 (71.1)	< 0.001
**Felt down or sad**^**a**^	1081 (37.5)	987 (60.7)	< 0.001

*Weighted percentages based on non-missing values. ^a^Argentina: In the past 12 months; Palestine: In past 2 weeks

**Table 2:** Prevalence of smoking behaviors among adolescents 14–17 years old from Argentina and the oPt, by gender (N = 4524)

	Girls (***N*** = 2310)			Boys (***N*** = 2214)		
	Argentina ***N*** = 1575 N (%)^**a**^	oPt ***N*** = 735 N (%)^**a**^	***p***-value	Argentina ***N*** = 1322 N (%)^**a**^	oPt ***N*** = 892 N (%)^**a**^	***p***-value
**Smoked cigarettes in past month**^**a**^	498 (33.1)	52 (7.1)	< 0.001	483 (36.5)	352 (39.6)	0.326
**Number of days smoked in past month**^**b**^						
1–2 days	265 (51.7)	37 (71.1)	0.166	204 (39.4)	90 (25.4)	< 0.001
3–5 days	94 (19.5)	7 (13.2)		85 (18.4)	47 (13.5)	
6–9 days	51 (11.2)	2 (3.9)		55 (11.3)	31 (8.8)	
10–19 days	37 (7.2)	3 (6.1)		64 (14.6)	48 (13.6)	
20–29 days	18 (3.7)	2 (3.9)		36 (7.8)	29 (8.3)	
All 30 days	33 (6.9)	1 (1.8)		39 (8.6)	107 (30.4)	
**Cigarettes smoked on days smoked**^**b**^						
< 1 cig/day	306 (60.9)	47 (90.5)	< 0.001	248 (48.0)	181 (51.3)	0.001
1 cig/day	80 (17.3)	4 (7.5)		90 (20.5)	35 (10.1)	
2–10 cig/day	101 (20.0)	1 (2.0)		121 (27.1)	116 (33.1)	
> 10 cig/day	10 (1.9)	0		20 (4.4)	20 (5.6)	
**Able to stop smoking if wanted to**^**b**^	452 (92.3)	48 (92.3)	1.000	439 (92.1)	264 (74.9)	< 0.001
**Age of first cigarette** ^**b**^						
< 8	39 (8.3)	6 (11.6)	0.344	44 (8.1)	28 (8.0)	< 0.001
8–9	17 (2.8)	1 (1.9)		21 (4.7)	34 (9.6)	
10–11	44 (7.9)	8 (16.1)		51 (10.0)	73 (20.6)	
12–13	197 (41.6)	16 (32.1)		197 (41.8)	126 (36.0)	
14–15	170 (33.3)	15 (30.2)		136 (29.6)	83 (23.5)	
16+	31 (6.0)	4 (8.0)		33 (5.9)	8 (2.3)	

^a^Weighted percentages of total sample ^b^ Weighted percentages of smokers

**Table 3:** Prevalence of exposure to violence among adolescents 14–17 years old from Argentina and the oPt, by gender (N = 4524)

	Girls (N = 2310)			Boys (N = 2214)		
	Argentina N = 1575 N (%)^**a**^	oPt N = 735 N (%)^**a**^	***p***-value	Argentina N = 1322 N (%)^**a**^	oPt N = 892 N (%)^**a**^	***p***-value
**Verbal violence**						
From family	515 (32.6)	355 (48.4)	< 0.001	275 (20.1)	419 (47.1)	< 0.001
**Physical violence**						
From family	270 (16.8)	362 (49.3)	< 0.001	145 (10.6)	503 (56.4)	< 0.001
From non-family	183 (12.1)	726 (98.8)	< 0.001	319 (26.2)	864 (96.8)	< 0.001
From family or non-family	391 (24.8)	732 (99.6)	< 0.001	418 (33.3)	880 (98.6)	< 0.001
**Verbal or physical violence**						
From family	569 (35.9)	456 (62.2)	< 0.001	315 (23.2)	595 (66.8)	< 0.001
From family or non-family	651 (41.2)	732 (99.6)	< 0.001	528 (41.1)	881 (98.7)	< 0.001

^a^Weighted percentages based on non-missing values

Multivariate logistic regression models included exposure to violence as the independent variable. Separate models were constructed for Argentina and for the West Bank, oPt samples and assessed the effect of exposure to violence (separate models for each individual verbal and physical violence variable independently, and the aggregated variables) on cigarette smoking in the past month (Table 4). Covariates were selected on the basis of those related to smoking behavior in the literature (gender, age, highest education level of primary caregiver, employment status of primary caregiver, parents’ smoking status, siblings’ smoking status, teachers’ smoking status, self-reported health status, felt anxious, and felt sad). We estimated adjusted odds ratios and 95% confidence intervals. For each research site, a second set of models were presented for girls and for boys separately (Table 5).

**Table 4:** Exposure to different forms of violence and odds of smoking among adolescents 14–17 years old, stratified by Country, Jujuy, Argentina in 2006 and West Bank oPt in 2008

	Argentina		oPt	
	OR^**a**^ (95% CI)	***p***-value	OR^**a**^ (95% CI)	***p***-value
**Verbal violence**				
From family	1.4 (1.1.-1.6)	0.002	1.4 (1.0–1.9)	0.047
**Physical violence**				
From family	1.2 (0.9–1.6)	0.169	1.3 (0.9–1.7)	0.125
From non-family	2.3 (1.8–2.9)	< 0.001	1.2 (0.5–2.8)	0.725
From family or non-family	1.8 (1.4–2.2)	< 0.001	0.8 (0.2–3.5)	0.773
**Verbal or physical violence**				
From family	1.3 (1.1–1.5)	0.009	1.4 (1.0–1.9)	0.034
From family or non-family	1.6 (1.3–1.8)	< 0.001	0.6 (0.1–2.9)	0.543

^a^Odds ratios adjusted for gender, age, provider highest education level, father or mother smoke, sibling smokes, teachers smoke in class, self-perceived health status, felt anxious, felt sad

**Table 5:** Exposure to different forms of violence and odds of smoking among adolescents 14–17 years old, stratified by Country and Gender, Jujuy, Argentina in 2006 and West Bank (oPt) in 2008

	Argentina				oPt			
	Girls		Boys		Girls		Boys	
	OR^**a**^ (95% CI)	***p***-value	OR^**a**^ (95% CI)	***p***-value	OR^**a**^ (95% CI)	***p***-value	OR^**a**^ (95%CI)	***p***-value
**Verbal violence**								
From family members	1.3 (1.0–1.7)	0.030	1.5 (1.1–2.0)	0.026	2.2 (1.1–4.4)	0.021	1.2 (0.8–1.7)	0.310
**Physical violence**								
From family	1.3 (0.9–1.9)	0.226	1.1 (0.8–1.4)	0.495	2.0 (1.1–3.6)	0.030	1.1 (0.8–1.6)	0.474
From non-family	2.5 (1.7–3.8)	< 0.001	2.2 (1.6–3.0)	< 0.001	1.0	-^b^	1.0 (0.4–2.6)	0.999
From family or non-family	1.6 (1.1–2.3)	0.011	2.0 (1.5–2.7)	< 0.001	1.0	-^b^	0.6 (0.1–3.4)	0.599
**Verbal or physical violence**								
From family	1.2 (1.0–1.6)	0.098	1.3 (1.0–1.8)	0.061	2.5 (1.2–5.4)	0.018	1.3 (0.9–1.8)	0.232
From family or non-family	1.5 (1.2–1.9)	0.003	1.7 (1.3–2.4)	0.002	1.0	-^b^	0.4 (0.1–2.8)	0.386

^a^Odds ratios adjusted for all risk factors ^b^Violence exposure occurred in 100% of cases

## Results

Table 1 compares the sociodemographic, health related and contextual smoking characteristics of respondents from Argentina and the West Bank, oPt. The Argentinian sample had a higher proportion of girls (54.9% vs. 45%). A higher percentage of respondents from Argentina reported believing that they would smoke if a friend offered a cigarette (38.6% vs. 18.2%) and that they would smoke a cigarette in the next year (44.5% vs. 21.9%). A higher percentage of respondents in the West Bank sample were of younger age (14–15 years) (83.5% vs. 68.8%), had a parent or primary provider who was not employed (16.1% vs. 11.5%), had parents who smoked (56.8% vs. 47.8) and reported that their health was very good or excellent (82.9% vs. 41%). However, a greater proportion of Palestinian youth reported feeling tense, nervous or anxious (71.1% vs. 30.4%) or feeling down or sad (60.7% vs. 37.5%).

### Smoking behavior

The current smoking prevalence was 34.6% in Argentina and 24.9% in the oPt (*p* < 0.001) (data not shown in table). The prevalence of smoking behaviors by country and by sex is shown in Table 2. Argentinian girls had almost five times the rate of cigarette smoking in the past month compared with Palestinian girls (33.1% vs. 7.1%, *p* < 0.001). The difference in rates for boys between the two samples was not significant (36.5% vs. 39.6%, *p* 0,326). There was no significant difference in the number of smoking days in the past month for girls in the two sites, but a higher proportion of Argentinian girls reported smoking more than 10 cigarettes on the days that they smoked (1.9% vs. 0%). However, compared with Argentinian boys, a greater percentage of Palestinian boys reported smoking during all 30 days of the month (30.4% vs. 8.6%) and smoking more than 10 cigarettes on the days that they smoked (5.6% vs. 4.4%). The perception of being able to stop smoking was more prevalent among Argentinian boys (92.1% vs. 74.9%) and a greater percentage of Argentinian boys, initiated smoking after 16 years of age (5.9% vs. 2.3%), compared with Palestinian boys.

### Exposure to violence

Table 3 shows the prevalence of exposure to violence by sex and by site. Palestinian girls and boys reported greater exposure to verbal and physical violence from family members and from nonfamily members, compared with Argentinians. The rate of exposure to any physical and verbal violence was almost universal and more than double for Palestinian girls (99.6% vs. 41.2%) and boys (98.7% vs. 41.1%) compared with Argentinians. Exposure to physical violence from nonfamily members was eight times greater for Palestinian girls (98.8% vs. 12.1%, *p* < 0.001) and almost four times greater for Palestinian boys (96.8% vs. 26.2%, p < 0.001), compared to Argentinian youth. Exposure to verbal violence from family members was also less common among youth in Argentina compared to oPt.

### Evaluating the Association of Exposure to violence and smoking

In multivariate logistic regression models without stratification by sex, exposure to verbal violence increased the odds of smoking in the past month among youth in both samples (Adjusted OR 1.4). Exposure to physical violence from non-family members was a significant risk factor only for Argentinian respondents (Adjusted OR 2.3) (Table 4).

Nearly all Palestinian girls reported exposure to violence from nonfamily members. As a result, models stratified by gender for the Palestinian sample yielded non convergent results for variables related to nonfamily members. Exposure to verbal (Adjusted OR 2.2; 95% CI = 1.1–4.4) or physical (Adjusted OR 2.0; 95% CI = 1.1.-3.6) violence from family members increased the odds of smoking among Palestinian girls. No forms of violence were significantly associated with smoking among Palestinian boys. Among Argentinian girls, verbal violence from family members (Adjusted OR 1.3; 95% CI = 1.0–1.7) and physical violence from nonfamily members (Adjusted OR 2.5; 95% CI = 1.7–3.8) increased the odds of smoking, with similar results for boys (Adjusted ORs 1.5 to 2.2) (Table 5).

## Discussion

Data from this study showed a direct association between exposure to violence and cigarette smoking among youth from two different areas, with country and gender specific variations. Results highlight the near universal exposure to violence among Palestinian youth, that may be related to higher intensity over a long period of time, of armed conflicts, with collective and individual repercussions [44]. Smoking was associated with some form of violence among Argentinian boys and girls but only among girls in the Palestinian sample. Gender-based patterns emerged on the basis of exposure to family violence, and among Palestinian girls who have low rates of cigarette use, smoking associated with violence exposure could acquire greater relevance in the future as global marketing, social media influences, and weakening of traditional social norms may promote the diffusion of smoking behavior [12, 30, 50–52].

Potential explanatory pathways for country and gender differences should be examined through empirical studies examining the role of social norms and/or differences in coping strategies. The following possible explanations for our findings are anchored in the coping theory [13], the social learning theory [14] and the tension reduction theory [15]. Girls in the two study sites could use smoking as a form of rebelliousness against social restrictions regarding tobacco use. In Argentina, girls who reported more egalitarian gender role attitudes had higher odds of smoking [12]. In the Middle East, social capital and women’s autonomy have been identified as contributing to women’s tobacco use [53]. Traditional gender norms commonly view women smoking as inappropriate, while men smoking is considered normative [54]. On the other hand, increased social acceptance of women’s smoking is attributed to a general pattern of liberalization of norms concerning women’s behavior [55]. Results for boys in the two areas could be explained by differences in the strategies used for coping with stressful situations. A study in Argentina found that boys are characterized by the use of problem avoidance coping strategies [56]. On the other hand, a cross-country study showed that Palestinian youth utilize more strategies based on problem solving and social action, than German or Australian youth. Argentinian boys may respond to violence exposure by smoking cigarettes due to the use of avoidance or emotion focused strategies, while Palestinian boys may be more prone to resort to problem solving strategies using actions or behaviors that do not involve cigarette smoking [29, 30]. Differences in coping behaviors across domains such as individualistic versus collectivist cultural orientations, and the use of tension reduction or spiritual support have been also ascertained [28, 30, 57–60]. Likewise, gender-based differences in coping strategies have been identified, for example increased use of emotionally focused strategies among girls versus disengagement strategies among boys [61, 62].

The results that refer to smoking behavior validate reported gender differences showing very low cigarette smoking prevalence among Palestinian girls [3, 63]. The prevalence of current cigarette smoking is similar for Argentinian and Palestinian boys. However, other smoking patterns are indicative of potential health risks for Palestinian boys as a greater proportion initiate smoking at an early age and smoke on a daily basis.

Palestinian girls and boys were exposed to higher rates of violence from family and nonfamily sources compared with Argentinians. Furthermore, nearly all of youth in the West Bank, oPt reported exposure to verbal and physical violence from nonfamily members. The level of reported exposure to violence among youth in the West Bank, oPt is extremely high even when compared across worldwide locations [64, 65]. The fact that associations with smoking were found with youth from Jujuy where there is no ongoing external conflict would support the existence of both common and area-specific explanatory pathways, anchored in youth’s individual and contextual experiences [66].

Other published findings (Table 6) have shown positive associations among youth who are the subject of, or who are victims of bullying, involved in physical fights, or witnesses of violence [21–27]. Regarding gender differences, one study showed that boys who witnessed a violent death were significantly more likely to smoke compared with girls [25], but another study showed no significant differences between girls and boys who were involved in bullying or a physical fight [23]. None of these studies asked about family violence nor were conducted in areas with long-term armed conflicts.

**Table 6:** World-wide Youth studies assessing the relationship between exposure to violence and smoking behavior

City/country school-based studies				
City or Country, Reference	Setting/Year	Sample Size	Violence Exposure	Association with Smoking
Beijing, China (Hazemba et al., 2008 [21])	Global School-Based Health Survey (GSHS)/2003	2348 middle school students	Bullying	OR = 1.09 students who smoked more likely to have been bullied
Malawi (Kubwalo et al., 2013 [22])	Malawi School-Based Student Health Survey/2009	2264, 13–15 yrs. old	Bullying	Students who smoked more likely of being bullied OR = 3.97
U.S/National (Hertz et al., 2015 [23])	Youth Risk Behavior Survey/School-based/2011	13,846, grades 9–12 yrs. old	Bullying	Being bullied significantly associated with cigarette use among girls and boys Adjusted ORs 1.7 to 2.3
Chile (Page 2009 [24])	Global School-Based Health Survey	8131, 13 to 15 yrs. old	Being involved in a fight in the prior 12 months	Cigarette smoking positively associated with having been involved in a fight
U.S//Boston (Pabayo, Molnar and Kawachi, 2013 [25])	School-based	High school students	witnessed a violent death	Witnessing a violent death associated with smoking among boys; girls not significant
**Cross-country school-based study**				
6 Western Pacific Countries—all Pacific Islanders (Yang et al., 2017 [26])	School-based	6377 youth aged 13–15 yrs. old	Physical fights	Significant association of fights with smoking
**Cross country community- based study**				
Five urban sites: Baltimore, New Delhi, Johannesburg, Ibadan, and Shanghai (Mmari et al., 2014 [27])	Community-based/2011-2013	2320 youth, 15–19 yrs. old	Witnessing community violence	Associated with ever smoking in 2 sites

This study has several limitations. The sampling and data collection strategies for the two countries were comparable. However, the indicators used to assess exposure to violence were not identical, reflecting site specific contextual characteristics. The cross-sectional nature of the data does not support the establishment of causal inferences regarding smoking and violence. Likewise, the role of covariates like anxiety or sadness in causal pathways could not be assessed. Individual and collective exposure to violence may have a different impact on smoking behavior that could not be evaluated with the measures used. Differences between personal involvement in violence and being a witness to violence should also be addressed in future studies. Lastly, the data are now 10 years old, although the social conditions that lead to violence and poverty in both the West Bank, oPt and in Jujuy have not significantly improved. Despite these limitations, our results contribute to the scarce literature on cross-country analysis of violence and cigarette smoking by gender in school youth populations.

## Conclusion

This study showed a significant association of exposure to violence with smoking behavior after adjusting for individual factors among youth from two areas of the world with specific contextual characteristics. This knowledge may inform and increase the impact of targeted smoking prevention efforts. Social sciences research has the potential for explaining context specific evidence, through psychological, historical, and cultural analysis.

## Data Availability

The datasets used and/or analyzed during the current study are available from the corresponding author upon request.
